# Characterization, localization, and seasonal changes of the sucrose transporter FeSUT1 in the phloem of *Fraxinus excelsior*

**DOI:** 10.1093/jxb/erv255

**Published:** 2015-05-28

**Authors:** Soner Öner-Sieben, Christine Rappl, Norbert Sauer, Ruth Stadler, Gertrud Lohaus

**Affiliations:** ^1^Molekulare Pflanzenforschung/Pflanzenbiochemie (Botanik), Bergische Universität Wuppertal, Gaußstraße 20, D-42119 Wuppertal, Germany; ^2^Lehrstuhl Molekulare Pflanzenphysiologie Department Biologie, Universität Erlangen-Nürnberg, Staudtstrasse 5, D-91058 Erlangen, Germany

**Keywords:** Immunolocalization, *Fraxinus excelsior*, heterologous expression, phloem loading, sucrose transporter.

## Abstract

Trees are generally assumed to be symplastic phloem loaders. A typical feature for most wooden species is an open minor vein structure with symplastic connections between mesophyll cells and phloem cells, which allow sucrose to move cell-to-cell through the plasmodesmata into the phloem. *Fraxinus excelsior* (Oleaceae) also translocates raffinose family oligosaccharides in addition to sucrose. Sucrose concentration was recently shown to be higher in the phloem sap than in the mesophyll cells. This suggests the involvement of apoplastic steps and the activity of sucrose transporters in addition to symplastic phloem-loading processes. In this study, the sucrose transporter FeSUT1 from *F. excelsior* was analysed. Heterologous expression in baker’s yeast showed that FeSUT1 mediates the uptake of sucrose. Immunohistochemical analyses revealed that FeSUT1 was exclusively located in phloem cells of minor veins and in the transport phloem of *F. excelsior*. Further characterization identified these cells as sieve elements and possibly ordinary companion cells but not as intermediary cells. The localization and expression pattern point towards functions of FeSUT1 in phloem loading of sucrose as well as in sucrose retrieval. FeSUT1 is most likely responsible for the observed sucrose gradient between mesophyll and phloem. The elevated expression level of *FeSUT1* indicated an increased apoplastic carbon export activity from the leaves during spring and late autumn. It is hypothesized that the importance of apoplastic loading is high under low-sucrose conditions and that the availability of two different phloem-loading mechanisms confers advantages for temperate woody species like *F. excelsior*.

## Highlight

In *Fraxinus excelsior* the sucrose transporter FeSUT1 is located in the phloem and involved in apoplastic sucrose loading and retrieval. The expression of *FeSUT1* is higher under low sucrose conditions.

## Introduction

Deciduous trees are highly integrated systems of different source tissues and of competing carbohydrate sinks. Whereas the major sinks in herbaceous annuals are generative organs and roots, the major sinks in trees include wood tissue that exhibits annual cycles of carbohydrate storage and remobilization for growth. Plants produce a variety of sugars and while one portion of the soluble carbohydrates is synthesized for carbon storage purposes another portion mainly functions as transport vehicles for energy and carbon. Indeed, as much as 50–70% of the photosynthetically fixed CO_2_ in mature leaves of herbal plants is exported from the leaf during the light period (Riens *et al.*, 1994). Most of the carbohydrates move to sink tissues through the phloem. Sugar production, phloem loading, and delivery through the phloem are different options that can be regulated to cope with environmental and seasonal changes. This is especially true for woody species because of their long lifespan and exposure to potentially extreme climate changes during annual cycles in temperate forests (De Schepper *et al.*, 2013). However, the regulation processes involved in carbon partitioning in trees are far from understood because most research regarding phloem loading as a regulatory step in carbohydrate delivery has been performed in herbaceous species.

### Mechanisms of phloem loading

There are currently three major mechanisms of phloem loading discussed:

(i) The active apoplastic phloem-loading mechanism is associated with a closed minor vein type (type 2) (Gamalei, 1991). Typical for closed minor veins are sieve element-companion cell complexes (SE-CCC), which are not connected to the neighbouring cells by plasmodesmata. Sucrose diffuses symplastically through plasmodesmata from mesophyll cells (MCs) into bundle sheath cells (BSCs) or phloem parenchyma cells (Schulz, 2015). From these cells sucrose will be released to the apoplast by SWEET proteins (Chen *et al.*, 2012) and is taken up into phloem companion cells (CC) by proton-coupled transporters (Giaquinta, 1977; Riesmeier *et al.*, 1994; Stadler *et al.*, 1995; Sauer, 2007). This process results in a higher concentration of sucrose in the SE-CCC than in the surrounding cells.

(ii) The active symplastic phloem-loading mechanism, known as the ‘polymer-trap’ mechanism, is associated with an open minor vein anatomy (type 1) (Gamalei, 1991), the presence of so-called intermediary cells (ICs), and the transport of raffinose family oligosaccharides (RFOs). Sucrose moves from BSCs through branched plasmodesmata into the ICs, which are specialized companion cells in the minor vein phloem. Within the ICs sucrose and galactinol are used as substrates for the synthesis of RFOs (Turgeon *et al.*, 1993; Voitsekhovskaja *et al.*, 2009). According to the hypothesis, the plasmodesmata fields at the BSC–IC interface act as filters that allow the disaccharide sucrose to pass, but reject the larger RFOs (Turgeon *et al.*, 1993; Liesche and Schulz, 2013). The movement of sucrose is maintained by the constant synthesis of RFOs (Voitsekhovskaja *et al.*, 2009) and the process results in high concentrations of RFOs in the phloem.

Minor veins that belong to the open type 1 group are heterogeneous with respect to the CCs. Hence, this group was further divided into type 1 (IC) and type I (CC) (Davidson *et al.*, 2011). Minor veins of type I (IC) contain ICs with abundant branched plasmodesmata on the IC side that are typical for RFO loaders, whereas the minor veins of type 1 (CC) contain ‘ordinary’ CCs. The CCs of type 1 (CC) have symmetrically branched or simple plasmodesmata on the MC or BSC interface with a higher frequency compared to CCs from type 2 minor veins (Davidson *et al.*, 2011).

(iii) The passive loading pathway is associated with minor vein type 1 (CC). Passive transport of sucrose into the phloem is characterized by a symplastic flux of sucrose that relies on a higher concentration of sugars in the MCs than in the SE-CCC. Owing to high plasmodesmatal connectivity between cells of the phloem and the surrounding cells in several tree species, it was assumed that trees are mostly passive phloem loaders (Davidson *et al.*, 2011). Indeed, plasmolysis and autoradiography experiments with ^14^C-sugars showed no accumulation of photoassimilates in minor veins of *Salix babylonica* L. (Turgeon and Medville, 1998), supporting the hypothesis of passive phloem loading in trees. In *Populus tremula x alba* (grey poplar), the expression of yeast invertase in the cell walls of transgenic plants did not inhibit phloem transport (Zhang *et al.*, 2014) and this result was discussed in relation to phloem loading of sucrose through plasmodesmata in poplar. However, in other tree species with an open minor vein type 1 (CC), like *Liriodendron tulipifera* or *Liquidambar styraciflua,* active apoplastic phloem loading has been shown to take place (Goggin *et al.*, 2001; Turgeon and Medville, 2004). In *Quercus robur* (oak), sucrose was more concentrated in the phloem sap than in the cytosol of MCs, which excludes the simple diffusion of sucrose in the direction of the phloem (Öner-Sieben and Lohaus, 2014).

Apparently, phloem-loading strategies are not necessarily predictable based on minor vein structure. Moreover, recent studies have shown that more than one phloem-loading mechanism could exist in parallel in a plant species or in a single minor vein, respectively (Braun and Slewinski, 2009; Voitsekhovskaja *et al.*, 2009; Gil *et al.*, 2011). Slewinski *et al.* (2013) have discussed that all plant species probably make use of multiple loading strategies.

### The role of sucrose transporters

Sucrose transporters (SUTs) play an important role in apoplastic phloem loading in higher plants (Sauer, 2007; Reinders *et al.*, 2012). Based on sequence homology and biochemical activity, SUTs have been divided into different groups, types, clades, or sub-families, depending on the authors chosen nomenclature (Sauer, 2007; Braun and Slewinski, 2009; Reinders *et al.*, 2012). Here, the nomenclature of Sauer (2007) is used. Proton-coupled Group I (monocot) and Group II (dicot) SUTs in the plasmamembrane of phloem cells mediate apoplastic phloem loading with sucrose or sucrose retrieval from the apoplast. Experimental evidence shows that suppression of these SUTs leads to several phenotypical aberrations in different species, such as stunted growth, sterility, and high accumulation of sugars in leaves (Riesmeier *et al.*, 1994; Gottwald *et al.*, 2000). Each angiosperm genome appears to have at least one sequence of the probably ancestral Group III transporter that contains an enlarged central cytoplasmatic loop (Reinders *et al.*, 2012). SUTs of Group IV are mainly found in the tonoplast of MCs, where they are apparently involved in sucrose efflux out of the vacuole into the cytosol (Endler *et al.*, 2006; Frost *et al.*, 2012; Schneider *et al.*, 2012).

Owing to the important role of SUT proteins in carbon allocation, *SUT* gene expression is tightly regulated on several levels (Ainsworth and Bush, 2011; Liesche *et al.* 2011). A great variety of factors, such as light, temperature, sugar concentrations, hormones, or pathogens, have an impact on *SUT* expression (Shakya and Sturm, 1998; Decourteix *et al.*, 2006; Gil *et al.*, 2011). The plasticity of phloem-loading activity has been demonstrated in response to defoliation in *Lolium perenne* (Berthier *et al.*, 2009). Most of the information on SUT localization, regulation, and function has been derived from studies on herbaceous plants. Only a few transporters have been described in trees so far, e.g. in *Juglans regia* L. (walnut), *Betula pendula* (birch), *Hevea brasiliensis* (para rubber tree), or poplar (Decourteix *et al.*, 2006; Wright *et al*., 2000; Tang *et al.*, 2010; Payyavula *et al.*, 2011).

*Fraxinus excelsior* has an open minor vein type 1 (IC) (Öner-Sieben and Lohaus, 2014), and it is thus categorized as an active symplastic phloem loader. It also contains two types of CCs in the minor veins, ICs and one or two ordinary CCs, similar to other symplastic loaders (Turgeon *et al.*, 1993; Voitsekhovskaja *et al.*, 2009; Öner-Sieben and Lohaus, 2014). Recent studies on *F. excelsior* revealed that, besides high amounts of RFOs, sucrose was present in high concentrations in the phloem sap as well. Moreover, a significant difference in sucrose concentration was observed, with higher concentration in the phloem than in the cytoplasm of MCs.

Based on these results it was assumed that phloem loading of sucrose needs to have an active component in addition to symplastic diffusion (Öner-Sieben and Lohaus, 2014).

In the present study, *FeSUT1* from *F. excelsior* was expressed in yeast and functionally characterized. Immunohistochemical analyses were performed on sections of leaf tissues to determine whether FeSUT1 may be involved in phloem loading. Moreover, the study focused on the tissue-specific sugar contents and *FeSUT1* expression in *F. excelsior* during the course of the year and under different light-dark regimes, especially in leaves.

## Materials and methods

### Plant material and experimental design

For the experiments, 30-year-old trees from a small forest near the campus (51° 14′ 42″ N, 7° 9′ 5″ E) and 3-year-old saplings that were grown in 5L pots in an open greenhouse were used. Pots were filled with soil that was dug out from a mixed forest (near Wuppertal, Germany). Plant material was obtained from a nursery (Baumschule Selders, Hilden, Germany). Sidewalls of the greenhouse were made of wire to allow free airflow. Experiments were designed as follows: (A) For immunolocalization of FeSUT1, leaves were taken from three 30-year-old trees. Leaves were taken at about 2–3 m height after about 6h of daylight. (B) Tissue-specific samples were taken from three 3-year-old saplings grown in 5L pots. Samples were taken in winter (9 January 2013; T = −4°C), in spring (25 April 2013; T = 18°C), and in summer (25 July 2013, T = 29°C), always in the second half of the light period. Samples were stored at −80°C until analysis. (C) For seasonal monitoring, leaves of three 30-year-old trees were sampled every month over a course of 3 years from 2009 to 2012. Leaves were taken at about 2–3 m height after 6–8h of daylight and stored at −80°C until analysis. (D) For light and dark experiments, leaves were taken from three 3-year-old saplings. Experiments were conducted on the 18–20 July 2012 with plants in the greenhouse described above. The first set of leaves was taken in the second half of the light period. After the end of the illumination period three plants were translocated in a completely dark room. Leaf samples were taken after 5h, 17h, and 43h of darkness.

### Expression of *FeSUT1* in *Saccharomyces cerevisiae* and uptake experiments

The shuttle vector NEV-E (Sauer and Stolz, 1994) was used for heterologous expression of *FeSUT1* in *Saccharomyces cerevisiae*. *FeSUT1* was cloned by reverse transcription PCR and RACE from leaves of *F. excelsior* (Öner-Sieben and Lohaus, 2014). To enhance the expression, the 5′-untranslated region of *PmSUC2* (5′-AAGCTTGTAAAAGAA-3′; Gahrtz *et al.*, 1994; Sauer and Stolz, 1994) was introduced into the full-length cDNA sequence of *FeSUT1* that was subsequently cloned into the *Eco*RI site of the vector. The yeast strain SEY2102 (Emr *et al.*, 1983) was transformed with plasmids harbouring inserts in sense or antisense orientation. Uptake was analysed as described (Gahrtz *et al.*, 1994).

### Preparation of plasma membranes and SDS-polyacrylamide gel electrophoresis

Isolation of yeast plasma membrane proteins and SDS-polyacrylamide gel electrophoresis are described elsewhere (Laemmli, 1970).

### Production and purification of anti-FeSUT1 antiserum

A region within the central loop of the protein was chosen for immunization. The corresponding peptide sequence QAEPPENIGHGVVK was synthesized, coupled to keyhole limpet haemocyanin, and used to immunize three rabbits (Pineda Antikörperservice, Berlin, Germany). The crude anti-FeSUT1 antisera were purified by adsorption to the synthetic FeSUT1 peptide coupled to keyhole limpet haemocyanin, which had been immobilized on nitrocellulose membranes (Sauer and Stadler, 1993; Schmitt *et al.*, 2008).

### Tissue preparation, immunohistochemistry, and fluorescence microscopy

The plant tissue was immersed in fixative (75% (v/v) ethanol p.A., 25% (v/v) glacial acidic acid) for 90min at 4°C. Embedding was carried out as described earlier (Stadler and Sauer, 1996; Schmitt *et al.*, 2008). All immunohistochemical analyses were performed with purified Anti-FeSUT1 antiserum (diluted 1:4 in blocking buffer). Anti-rabbit IgG Alexa Fluor®488 (Thermo Fischer Scientific, Waltham, MA, USA) was used as secondary antibody. Callose was stained with 0.1% aniline blue (Water Blue; Fluka, Buchs, Switzerland) in TBS for 5min. Aniline blue fluorescence was detected with an excitation light of 365nm using a conventional fluorescence microscope (Axioskop; Carl Zeiss, Jena, Germany). Overlay images of Alexa Fluor®488 and aniline blue fluorescence were created using the Adobe Photoshop CS6 software. All other fluorescence and phase-contrast images were created at the confocal laser microscopes TCS SPII and TCS SP5 (Leica Microsystems, Wetzlar, Germany).

### Extraction of soluble carbohydrates from tissue and sugar analyses

Tissues were ground to a fine powder with pestle and mortar in liquid nitrogen. Water-chloroform-methanol extracts were prepared according to Riens *et al.* (1991). Sugars and sugar alcohols in tissue extracts were analysed by HPLC according to Öner-Sieben and Lohaus (2014).

### RNA isolation and quantitative reverse transcription PCR

RNA from different tissues was isolated using a modified protocol from Chang *et al.* (1993). Synthesis of cDNA was performed using the RevertAid^TM^ First Strand cDNA Synthesis Kit (Thermo Fisher Scientific, St. Leon-Rot, Germany) with oligo(dT)_18_ primers. Quantitative real-time PCR (qPCR) analyses were performed using a Maxima SYBR Green qPCR Master Mix, (Thermo Fisher Scientific) and a Mx3005P qPCR System (Agilent Technologies, Inc., Waldbronn, Germany) with the software MxPro Version 4.10 (cycle protocol: 10 s 95°C denaturation; 60°C combined annealing and elongation). Slopes of standard curves of 2-fold dilutions were used to determine the efficiencies of PCR reactions. The following primer sets with corresponding amplicon lengths of 235bp (actin) and 127bp (*FeSUT1*) were used: *Actin forward*: AGA GAT TCC GTT GCC CAG AA; *Actin reverse*: GCC ACA ACC TTA ATC TTC ATG C; *FeSUT1 forward*: GCT CTC CTT GTT GAC TCC; *FeSUT1 reverse*: ATT GTC ACT GTA GTA GCC A. Relative initial mRNA concentrations of *FeSUT1* (GenBank accession number: KF736981) in *F. excelsior* were estimated by normalizing expression levels to the housekeeping gene actin (AM063027), which was used as a control gene in previous studies (Chen *et al.*, 2013; Li *et al.*, 2013). The first sample of each experiment was used as a calibrator, which was set to one, and further samples are given as relative expression levels to the calibrator.

### Statistical analysis

To estimate the probability of the mean values of the sampled tissues, a Student’s t-test with *P* = 0.05 was performed. Because values were obtained from samples of the same plants at different time points of a certain experiment or development a paired t-test was conducted.

## Results

### Characterization of FeSUT1

**Fig. 1.**
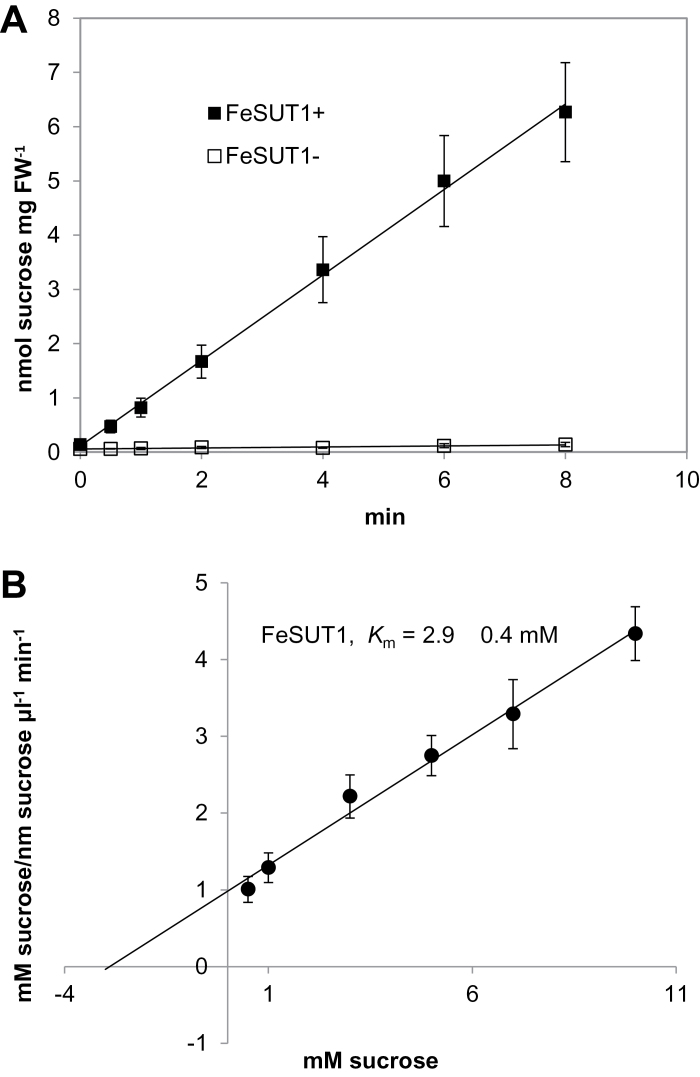
(A) Uptake of ^14^C-sucrose into SEY2102 yeast cells that expressed *FeSUT1*. The exogenous sucrose concentration was 1mM and measurements were conducted three times with three different transformants. (B) Hanes-Woolf plot for the calculation of the *K*_m_-value of FeSUT1. The initial sucrose concentrations to the reaction velocities are plotted against the sucrose concentrations.

Recent studies identified a Group II SUT from source leaves of *F. excelsior* (Öner-Sieben and Lohaus, 2014; Supplementary Fig. S1). This transport protein was examined to see if it is involved in the observed accumulation of sucrose in the phloem. To evaluate the properties of FeSUT1, the transporter was analysed in a yeast strain previously used for functional analysis of plant SUTs (Gahrtz *et al.*, 1994; Sauer and Stolz, 1994). The *FeSUT1* cDNA was cloned in sense and antisense direction into the shuttle vector NEV-E and the resulting constructs were used to transform the yeast strain SEY2102. Yeasts containing the sense plasmid constructs (FeSUT1+) transported ^14^C-sucrose at high rates whereas sucrose uptake in the negative control (FeSUT1−) was negligibly low (Fig. 1A). This result proved that FeSUT1 is in fact a functional sucrose transport protein. Kinetic analyses of ^14^C-sucrose uptake by yeast cells expressing *FeSUT1* revealed an apparent *K*_m_-value for sucrose of 2.9±0.4mM at pH 5.5 (Fig. 1B).

### Specificity of the anti-FeSUT1 antiserum

**Fig. 2.**
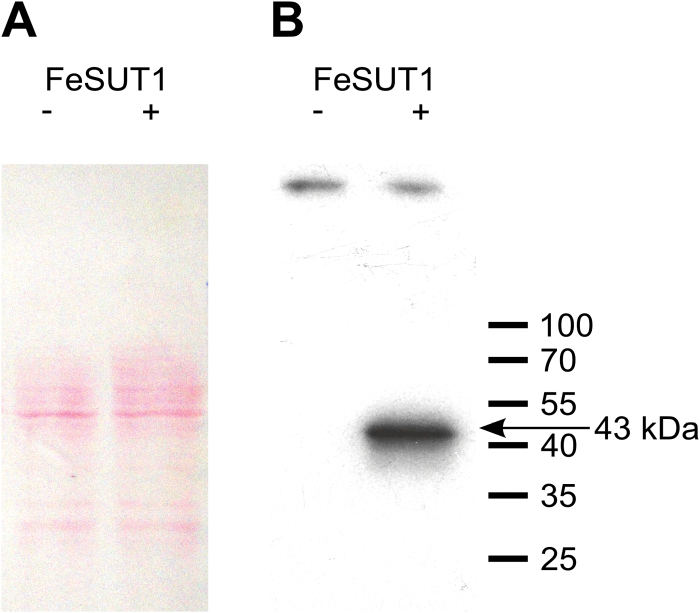
Western blot analysis of anti-FeSUT1 antiserum on isolated crude membrane protein extracts from yeast cells expressing *FeSUT1* in sense (lane +) and in antisense direction (lane −). (A) Successful blotting was verified by Ponceau S staining. (B) The anti-FeSUT1-antiserum showed a strong specific band at 43kDa on membrane protein extracts from transgenic yeast that expressed *FeSUT1* (lane +). The negative control showed no reactions (lane −). (This figure is available in colour at *JXB* online.)

Three polyclonal antisera against a part of the central loop of FeSUT1 were raised in rabbits. The specificity of the antisera was confirmed by western blot analyses using plasma membrane proteins from *FeSUT1*-expressing yeast cells. All three anti-FeSUT1-antisera resulted in similar signals, but the antiserum of rabbit 1 showed the strongest reaction. It recognized a polypeptide with an apparent molecular weight of 43kDa (*FeSUT1* in sense direction, *FeSUT1+* in Fig. 2B). No bands were present in the negative control (*FeSUT1* in antisense direction, *FeSUT1−* in Fig. 2B) although similar protein amounts were blotted (Fig. 2A). The apparent molecular weight (43kDa) was lower than the calculated molecular weight (54.2kDa) of FeSUT1, which was also shown for other lipophilic membrane proteins (Beyreuther *et al.*, 1980; Sauer and Tanner, 1984).

### Immunolocalization of FeSUT1

**Fig. 3.**
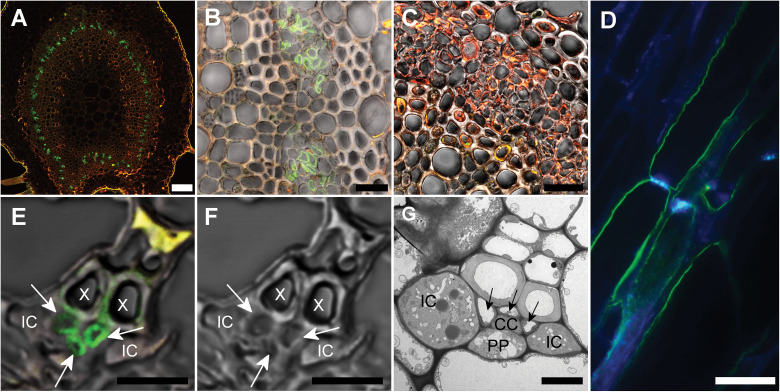
Localization of FeSUT1 in midribs and minor veins of *F. excelsior* leaves by fluorescence detection with anti-FeSUT1 antibody. Leaves were taken from ~30-year-old trees. Yellow or red staining shows autofluorescence of phenolics in lignified cells, green staining shows FeSUT1 localization. Specific anti-FeSUT1 antibody reaction is indicated by green Alexa Fluor® labelling. (A) Overview of a cross section of a leaf midrib treated with anti-FeSUT1-antiserum. Labelling was seen in the circularly arranged transport phloem. (B) Higher magnification of the transport phloem in the leaf midrib imaged in phase-contrast. Irregular shaped cells of the phloem were stained. (C) Section of the midrib incubated with antiserum prior to immunization as negative control imaged in phase-contrast. (D) Longitudinal sections of a vascular bundle of the midrib. Green staining shows FeSUT1 localization. Blue labelling shows detection of callose in sieve plates with aniline blue. (E) Phase-contrast image of the FeSUT1-localization in the minor veins of *F. excelsior*. Labelled cells (arrow) are SEs or CCs rather than ICs. (F) Same minor vein image without fluorescence light. (G) Electron micrograph of a minor vein of *F. excelsior* highlighting the regular arrangement of cells. SEs (arrowhead) and CCs are located in the central part of the minor veins whereas the ICs are laterally localized. Scale bars = (A) 100 µm; (B, C) 25 µm; (D) 20 µm; (E, F) 7.5 µm; (G) 5 µm. PP, phloem parenchyma cell; X, xylem.

To localize FeSUT1 on the cellular level, anti-FeSUT1-antiserum was used on sections from leaves of *F. excelsior*. Figure 3A, B shows cross-sections of the midrib of *F. excelsior* with anti-FeSUT1-antiserum-derived fluorescence signal in the transport phloem. Pre-immune serum was used as a negative control (Fig. 3C). Autofluorescence of cells containing phenolic compounds is shown in yellow or red.

To further confirm the identity of the labelled cells, longitudinal sections of the midrib of *F. excelsior* leaves were stained simultaneously with anti-FeSUT1 antibody and the callose/sieve plate-marker aniline blue. The resulting images showed that the cells that contained a sieve plate were also labelled with anti-FeSUT1-antiserum (Fig. 3D). However, in some of the cells labelled with anti-FeSUT1-antiserum no sieve plate was visible. For several species of Solanaceae it has been shown that some anti-SUT antibodies can bind to unknown, non-SUT epitopes in sieve elements (SEs) and this unspecific binding was not seen with purified antisera (Schmitt *et al.*, 2008). Based on that observation purified anti-FeSUT1 antisera were used for all experiments.

The immunolocalization of FeSUT1 in minor veins was investigated in cross sections of *F. excelsior* leaflets (Fig. 3E). In the minor vein of *F. excelsior* two types of CCs were present (Öner-Sieben and Lohaus, 2014). The minor vein contained two laterally positioned ICs with plasmodesmata fields that connect them to adjacent BSCs side. However, one or two ordinary CCs without this kind of plasmodesmata field were found in the central part of the minor vein. In addition three or four SEs were present in the central part of the minor vein adjacenct to xylem cells. After treatment with anti-FeSUT1 antibody, green fluorescence was seen in the inner part of the minor vein phloem. To further characterize the cell type, electron micrographs of minor veins from the same order were examined (Fig. 3G). Based on their position the anti-FeSUT1-antiserum-marked cells could be SEs and CCs but not the laterally positioned ICs.

### Tissue-specific sugar composition and expression of *FeSUT1* in winter, spring, and summer

**Fig. 4.**
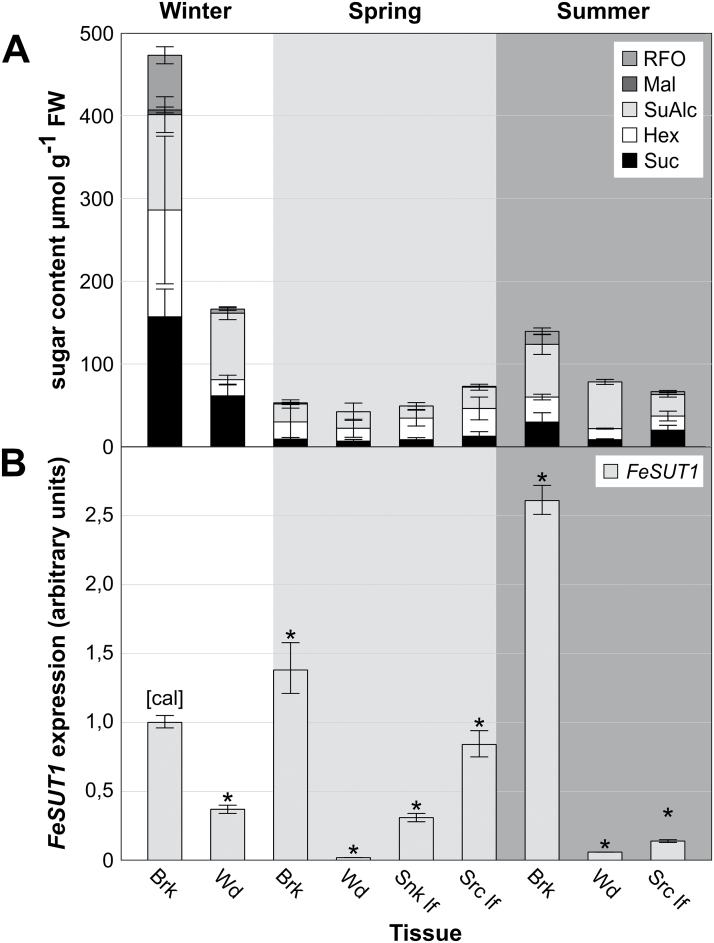
Tissue-specific sugar composition and expression of *FeSUT1* in *F. excelsior*. Samples were taken from three 3-year-old saplings grown in an open greenhouse. (A) Tissue-specific sugar and sugar alcohol content during winter, spring, and summer in *F. excelsior*. (B) Tissue-specific expression levels of *FeSUT1* during winter, spring, and summer. Expression levels of *FeSUT1* were normalized to actin and values are given as relative expression levels to the first sample of the measurement (calibrator [cal]). Student’s t-test was performed with *P ≤* 0.05 to test for significance of changes compared to calibrator [cal] (asterisk denotes significant difference). Brk, bark; Hex, hexoses; Mal, maltose; RFO, raffinose-oligosaccharide family sugars; Snk lf, sink leaf; Src lf, source leaf; SuAlc, sugar alcohols; Suc, sucrose; Wd, wood.

After the localization of FeSUT1 in phloem cells, environmental conditions that might influence *FeSUT1* expression were analysed. Sugar and sugar alcohol contents as well as mRNA levels of *FeSUT1* in different tissues of *F. excelsior* during different seasons were measured. The overall sugar and sugar alcohol contents were highest in bark [up to 500 µmol g^−1^ fresh weight (FW)] and wood in winter (Fig. 4A). Because data for sugar content and SUT expression refer to FW, and wood contains less symplast per gram FW than bark or leaves, the values for wood would be even higher if the data referred to the symplast. However, values were given per gram FW to ensure comparability with other published data (e.g. Ferner *et al.*, 2012). In the stem tissues the main sugars were sucrose, hexoses, and RFOs (Fig. 4A). Mannitol was by far the dominant sugar alcohol (more than 90% at the total content of sugar alcohols; data not shown). In spring, sugar and sugar alcohol contents were much lower (below about 80 µmol g^−1^ FW) in all tissues (bark, wood, sink leaves, and source leaves). Sink leaves and source leaves were defined by size and weight. ‘Source leaves’ in spring were identified as those 80–100% of the size and weight of mature leaves in summer, whereas ‘sink leaves’ had only about 10% of those values. In summer, sugar and sugar alcohol contents in bark and wood were higher than in spring (Fig. 4A) whereas the contents in source leaves were similar or slightly lower.

qPCR assays were normalized to actin, which was used as a control gene in other studies in *Fraxinus* (Chen *et al.*, 2013; Li *et al.*, 2013). Expression values of *FeSUT1* are given relative to the expression in the first collected bark sample in spring. Significant differences between the mean values of samples and the calibrator of each experiment were calculated using Student’s t-test with *P =* 0.05. *FeSUT1* expression showed a strong tissue-specific variation with highest expression levels in bark (Fig. 4B). In bark tissue the expression level of *FeSUT1* increased from winter to spring and was highest in summer. The lowest expression levels were found in wood during the whole season (Fig. 4B). The expression level of *FeSUT1* in source leaves decreased to about one sixth from spring to summer (Fig. 4B).

### Sugar composition and *FeSUT1* expression in leaves during growing season

**Fig. 5.**
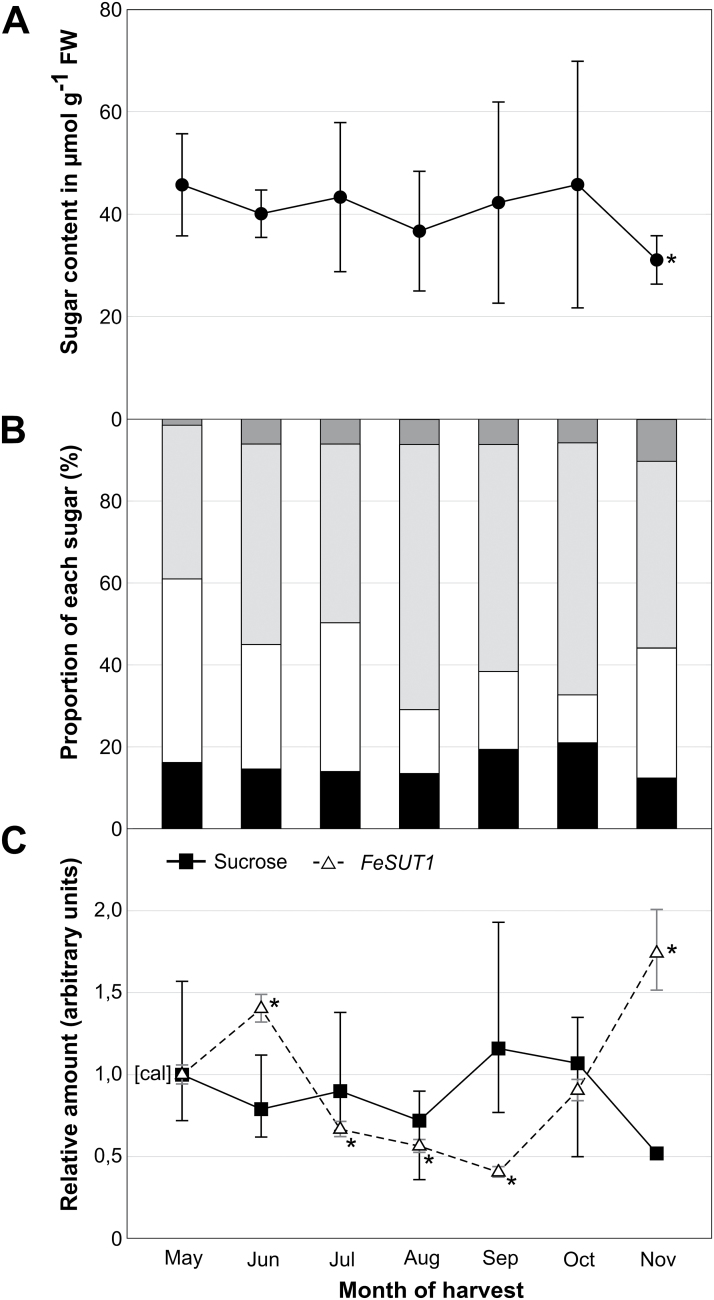
Sugar and sugar alcohol content as well as *FeSUT1* expression in leaves of *F. excelsior* during growing season. Samples were taken over a period of 3 years (2010–2012) from three individual trees from a 25- to 30-year-old forest. (A) Total sugar and sugar alcohol content in leaves. (B) Relative amounts of sugar and sugar alcohol at the total content. (C) Relative sucrose content (■, closed squares, continuous line) and relative expression of *FeSUT1* (Δ, open triangles, broken line). Expression levels of *FeSUT1* were normalized to actin and values are given as relative expression levels to the first sample of the measurement (calibrator [cal]). Student’s t-test was performed with *P ≤* 0.05 to test for significance of changes compared to calibrator [cal].

Leaves of ~30-year-old trees contained similar amounts of sugars and sugar alcohols between May and October (40 to 50 µmol g FW^−1^; Fig. 5A). Only in late autumn (November) did sugar content decrease. The high values of standard deviation (SD) are due to the fact that samples were taken from three different plants in their natural environment over a course of 3 years (always at the same time of the day (2 p.m.) and the same date of the month without regard to outside temperature, humidity, or hours of sunshine). The distribution of sugars and sugar alcohols varied during the growing season. Sugar alcohols (mainly mannitol) were the dominant component in leaves of *F. excelsior* for most of the year, followed by hexoses (hex) and sucrose (Fig. 5B). RFOs started with 1.5% in May and showed higher proportions in summer and autumn (Fig. 5B).

*FeSUT1* was expressed constantly during the whole growing season (Fig. 5C). Relative *FeSUT1* expression was higher in spring and early summer (May, June) and lower in summer and early autumn (July, August, September). Similar results were also shown for leaves of 3-year-old saplings (see Fig. 4B). In the second half of autumn, the expression level increased again (Fig. 5C). Sucrose content was similar in spring and summer and increased in early autumn (September and October). In leaves from November (leaves had shown strong signs of senescence), sucrose content decreased whereas expression levels of *FeSUT1* increased about 4-fold from September to November (Fig. 5C).

### Sugar composition and *FeSUT1* expression under different light-dark regimes

**Fig. 6.**
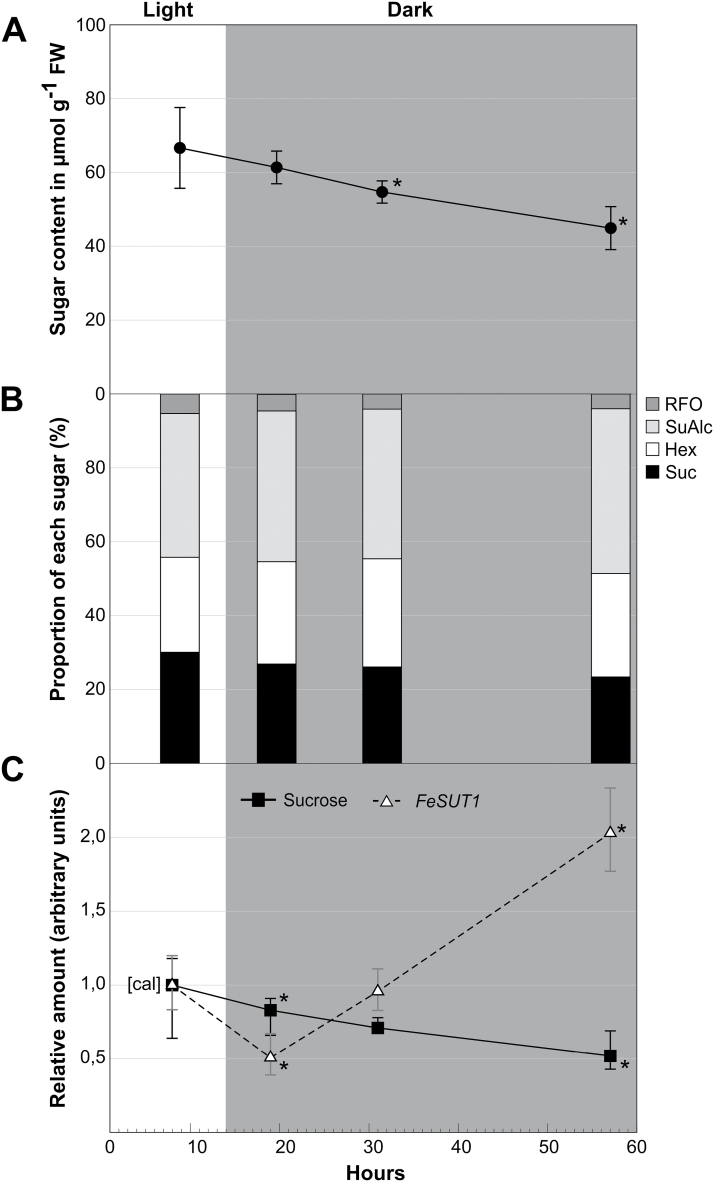
Sugar and sugar alcohol content as well as *FeSUT1* expression in leaves of *F. excelsior* in light and during a prolonged dark period. Experiments were performed with three 3-year-old saplings. Leaves were taken in the second half of the normal light period and after 5, 17, and 43h of continuous darkness. (A) Total sugar and sugar alcohol content in leaves. (B) Relative amounts of sugar and sugar alcohol at the total content. (C) Relative sucrose content (■, closed squares, continuous line) and relative expression of *FeSUT1* (Δ, open triangles, broken line). Expression levels of *FeSUT1* were normalized to actin and values are given as relative expression levels to the first sample of the measurement (calibrator [cal]). Student’s t-test was performed with *P ≤* 0.05 to test for significance of changes compared to calibrator [cal]. Hex, hexoses; Mal, maltose; RFO, raffinose-oligosaccharide family sugars; SuAlc, sugar alcohols; Suc, sucrose.

After transferring plants into continuous darkness, sugar and sugar alcohol content in leaves started to decline (Fig. 6A). Nevertheless, the sugar content in leaves after about 2 days of darkness was still two-thirds of the values found in illuminated leaves. The sugar and sugar alcohol composition also changed slightly during the long dark period. The proportion of sugar alcohol (mainly mannitol) increased whereas the proportion of RFOs and sucrose decreased (Fig. 6B). The expression of *FeSUT1* compared with sucrose content in *F. excelsior* is shown in Fig. 6C. Like the whole sugar content, sucrose content decreased after illumination stopped. In contrast, expression of *FeSUT1* increased strongly during the long dark period (Fig. 6C).

## Discussion

### Comparison of biochemical characteristics of FeSUT1 and other SUTs

*FeSUT1* described in the present study was isolated from source leaf RNAs of *F. excelsior* and identified as a Group II SUT (Öner-Sieben and Lohaus, 2014). Data about enzymatic features are primarily available for SUTs from herbs and according to the best available knowledge only one tree-specific Group II SUT from *Hevea brasiliensis* (HbSUT3; Tang *et al.*, 2010) has been characterized, in yeast. The apparent *K*_m_ value for sucrose of FeSUT1 was about 2.9mM and similar *K*_m_ values have been reported for other SUTs (Table 1). Group II SUTs have high substrate affinities with *K*_m_ values between 1 and 2mM (Table 1). Most Group III and IV SUTs have considerably lower substrate affinities and a greater variability regarding the measured *K*_m_ values (Table 1). FeSUT1 of *F. excelsior* showed great similarities, both structurally and biochemically, to AmSUT1 from *Alonsoa meridionalis*, which had a *K*_m_ value of 1.8mM. Both species are members of the order Lamiales and they are characterized as putative mixed apoplastic and symplastic phloem loaders (Knop *et al.*, 2001; 2004; Voitsekhovskaja *et al.*, 2009; Öner-Sieben and Lohaus, 2014).

**Table 1.** K*_m_ values of FeSUT1 for sucrose compared with values of other SUTs*

Species	Transporter	Group	*K*_m_	Protein-ID	Reference
*Hordeum vulgare*	HvSUT1	I	7.5 mM	*CAJ20123*	Weschke *et al.*, 2000
*Fraxinus excelsior*	FeSUT1	II	2.9±0.4 mM	AHB33870	this paper
*Asarina barclaiana*	AbSUT1	II	0.5 mM	AAF04294	Knop *et al.*, 2001
*Alonsoa meridionalis*	AmSUT1	II	1.8 mM	AAF04295	Knop *et al.*, 2004
*Arabidopsis thaliana*	AtSUC1	II	0.5 mM	CAA53147	Sauer and Stolz, 1994
*Arabidopsis thaliana*	AtSUC2	II	0.77 mM	CAA53150	Sauer and Stolz, 1994
*Medicago truncatula*	MtSUT1	II	1.7 mM	AFM28284	Doidy *et al.*, 2012
*Plantago major*	PmSUC2	II	1 mM	CAA53390	Gahrtz *et al.*, 1994
*Phaseolus vulgaris*	PvSUT1	II	8.5±0.7 mM	ABB30164	Zhou *et al.*, 2007
*Ricinus communis*	RcScr1	II	2 mM	CAA83436	Weig and Komor, 1995
*Solanum tuberosum*	StSUT1	II	1 mM	CAA48915	Riesmeier *et al.*, 1993
*Spinacia oleracea*	SoSUT1	II	1.5 mM	CAA47604	Riesmeier *et al.*, 1992
*Arabidopsis thaliana*	AtSUT2/AtSUC3	III	11.7 mM	CAB92307	Meyer *et al.*, 2000
*Plantago major*	PmSUC3	III	5.5±1.1 mM	CAD58887	Barth *et al.*, 2003
*Arabidopsis thaliana*	AtSUT4	IV	11.6±0.6 mM	AAL59915	Weise *et al.*, 2000
*Daucus carota*	DcSUT1a	IV	0.5 mM	CAA76367	Shakya and Sturm, 1998
*Hordeum vulgare*	HvSUT2	IV	5 mM	CAB75881	Weschke *et al.*, 2000
*Lotus japonicus*	LjSUT4	IV	12.9 mM	CAD61275	Flemetakis *et al.*, 2003
*Oryza sativa*	OsSUT2	IV	1.86±0.38 mM	BAC67163	Eom *et al.*, 2011
*Solanum tuberosum*	StSUT4	IV	6.0±1.2 mM	AAG25923	Weise *et al.*, 2000

### FeSUT1 is localized in the collection and transport phloem of *F. excelsior*

In order to function in phloem loading, *FeSUT1* should be expressed in phloem tissue. To verify this hypothesis, immunolocalization experiments were conducted on tissue sections of *F. excelsior*. The purified anti-FeSUT1-antiserum marked cells of both the transport and the collection phloem (Fig. 3). SUTs that were localized in the phloem have been described in several herbaceous species like *Plantago major*, *Arabidopsis thaliana*, *Solanum tuberosum, Alonsoa meridionalis*, and *Triticum aestivum* (Stadler *et al.*, 1995; Stadler and Sauer, 1996; Kühn *et al.*, 1997; Knop *et al.*, 2004; Aoki *et al.*, 2004; Schmitt *et al.*, 2008). In herbaceous plant species, like *P. major* and *A. thaliana*, SUTs have been identified to be localized in CCs (Stadler *et al.*, 1995; Stadler and Sauer; 1996). However, in solanaceous species, SUTs have been localized in both CCs as well as SEs (Kühn *et al.*, 1997; Schmitt *et al.*, 2008). Results for the woody species *F. excelsior* have clearly shown the localization of FeSUT1 in SEs (Fig. 3). Depending on plant species or plant tissues, SUTs could be found in different cell types of the phloem, SEs, and/or CCs, which may reflect the necessity to deploy transporters in response to local, temporal, or physiological needs of the plant.

SUTs are probably involved in the primary phloem loading with sucrose and in the retrieval of sucrose that leaks out of the sieve tubes into the lateral tissue (van Bel, 2003). While some of the leaked sucrose is used to supply the surrounding tissue with carbohydrates, most is transported back into the phloem to maintain the strong concentration gradient that drives the bulk flow (Minchin and Thorpe, 1987; Gould *et al.*, 2012). The loss of sucrose is probably due to diffusion and driven by the very high concentration itself (Kedem and Katchalsky, 1958). The sugar concentrations in the phloem sap of *F. excelsior* are also very high (sucrose 0.4M and RFOs 0.6M; Öner-Sieben and Lohaus, 2014), thus leakage of sucrose is likely. In addition the height of trees is normally about 100-fold larger than that of herbaceous plants (i.e. 30–50 m in relation to 30–50cm). Therefore, the distance between phloem loading and phloem unloading in trees is very often exceptionally large and retrieval of sucrose into the phloem to maintain the concentration gradient should be very important. In contrast, at least for small herbaceous plants, the retrieval function is not essential as shown for *Arabidopsis* (Srivastava *et al.*, 2008). In general, the SEs of the transport phloem are much larger than the CCs in this tissue (van Bel, 2003; De Schepper *et al.*, 2013). The direct contact of the large membrane surface of SEs with the apoplastic interface requires a set of uptake devices (van Bel, 2003). Retrieval of sucrose into the larger SEs can occur with a greater extent than into the smaller CCs, which would explain the presence of SUTs at the interface between SEs and apoplast (Fig. 3). Probably the localization of the sucrose carrier in the phloem is related to the relevance of sucrose retrieval for the respective species. Owing to the major importance of a retrieval mechanism for trees and its minor importance for herbaceous species, the sucrose carrier is localized to SEs in trees whereas it is localized predominately in CCs in herbaceous species. Maybe other factors like annual cycles also contribute to the different locations of SUTs in the transport phloem.

In addition to the transport phloem, FeSUT1 was also localized in the phloem cells of minor veins of *F. excelsior*, which confirmed the hypothesis that FeSUT1 functions in phloem loading. The structure of the minor veins of *F. excelsior* with two types of CCs (Öner-Sieben and Lohaus, 2014) is typical for RFO-translocating and putative symplastic phloem-loading species (Fisher, 1986; Hoffmann-Thoma *et al.*, 2001; Knop *et al.*, 2004). FeSUT1 was confined to SEs and possibly CCs but not to ICs (Fig. 3), even though ICs need to import a high amount of sucrose because the synthesis of RFOs for phloem transport occurs in ICs. Voitsekhovskaja *et al.* (2009) showed that the gene for stachyose-synthase *AmSTS1* was expressed exclusively in the ICs of *Alonsoa meridionalis.* Therefore, the fact that FeSUT1 was not localized in ICs indicates that the sucrose, which is necessary for RFO synthesis in ICs, was probably imported symplastically. FeSUT1 was located in SEs and probably CCs and in these cell types the transporter was most likely involved in active sucrose uptake from the apoplast. The SUT AmSUT1 has similar functions in *A. meridionalis* (Voitsekhovskaja *et al.*, 2009). That means two types of SE-CC complexes (SE-IC and SE-CC) within the same minor vein load or produce different carbohydrates and use contrasting mechanisms for their delivery into the phloem. It has been hypothesized that the phloem turgor regulates the conductance of the plasmodesmata leading into the CCs or ICs (Oparka and Prior, 1992; De Schepper *et al.*, 2013). Therefore phloem pressure could be one possibility regulating the extent of symplastic and apoplastic phloem loading.

### A shift in the phloem-loading mechanism occurs during growing season

Sugar transport is a dynamic process that is tightly regulated by environmental factors and by the physiological needs of the plants. Several factors, e.g. light (Kühn *et al.*, 2003), freeze-thaw cycles (Decourteix *et al.*, 2006), or salt stress (Noiraud *et al.*, 2000), have an impact on *SUT* expression. To get insight into possible mechanisms that regulate the expression of *FeSUT1*, qPCR assays were conducted and sugar contents were measured. The tissue-specific expression levels showed that *FeSUT1* was expressed in all tissues and throughout all measurements but it was predominantly expressed in bark (Fig. 4B). Bark contains the secondary phloem, which is primarily involved in assimilate transport from source to sink tissues. Interestingly, in winter the expression level of *FeSUT1* was also relatively high in wood indicating that transport processes are essential for basal physiological function during dormancy. *SUT* expression in wood has also been reported for other trees and it was even increased after freeze conditions in walnut (Decourteix *et al.*, 2006). During the transition from dormancy to the vegetative state in spring and summer the expression of *FeSUT1* increased in bark tissue while it declined in wood (Fig. 4B). A possible explanation could be that in spring the retrieval and transport of sucrose became more important and *FeSUT1* expression was up-regulated in the transport phloem. Seasonal redistributions of sucrose in stems have also been reported in other tree species, e.g. *Populus* (Sauter, 1988).

In leaves, *FeSUT1* expression was higher in spring than in summer (Fig. 4B). This indicates that apoplastic loading might be more important in spring than in summer. The analysis of the sugar and sugar alcohol content in the respective tissues gave another hint towards the presence of a possible shift of phloem-loading mechanisms. RFOs were nearly absent in spring but were detectable in bark and source leaf tissue in summer (Figs. 4A, 5B), meaning that the synthesis of RFOs was also up-regulated from spring to summer. The synthesis of RFOs is restricted to the ICs (Voitsekhovskaja *et al.*, 2009; Öner-Sieben and Lohaus, 2014) and corresponds to the ‘polymer trap’ hypothesis that sucrose diffuses symplastically into the ICs. The data suggest that apoplastic loading becomes less important when the polymer trapping mechanisms get established during the transition from the initial growth period in spring to a more vegetative state in summer.

### Apoplastic phloem loading increases during periods of lower sucrose content

The data indicate that *FeSUT1* expression is induced whenever the availability of sucrose is low. The overall sugar and sugar alcohol content as well as the sucrose content declined in November, when senescence of the leaves was far advanced (Fig. 5A, C). During this period the expression of *FeSUT1* reached its seasonal high (Fig. 5C). Deciduous species store high concentrations of soluble carbohydrates in the stem during winter as an antifreeze agent and as energy store for bud break in spring (Sauter, 1988). The elevated expression level of *FeSUT1* indicates an increased apoplastic carbon export activity from the leaves during late autumn.

Similar results were obtained for plants transferred into darkness (Fig 6). In leaves of these plants the overall sugar and sugar alcohol content as well as the sucrose content decreased during darkness (Fig. 6A, C). The decline in *F. excelsior* was less distinct than in herbaceous plants (Riens *et al.*, 1994). Even after about 2 days of permanent darkness, 68% of the sugar and sugar alcohol content measured in the second half of the light period was still present (Fig. 6A). Again, the expression of *FeSUT1* increased when sucrose availability declined (Fig. 6C).

The observed increase in *FeSUT1* expression indicates that apoplastic phloem loading is induced when the sucrose concentration is low. The symplastic movement of sucrose from the MCs or BSCs into the ICs of the phloem requires higher sugar concentration in the MCs than in the phloem. It is hypothesized that the sugar concentration in MCs falls below the critical concentration for this kind of movement under conditions of senescence or longer dark periods. Apoplastic phloem loading mediated by FeSUT1 could be necessary to maintain the transport of sucrose into the phloem. It has not been investigated so far if apoplastic phloem loading can occur efficiently in open minor veins. It would be interesting to study a possible plasmodesmatal closure between MCs and SE-CCCs during that time of the year.

Flexibility in processes of sucrose transport has also been shown for some other species, like *Cucumis melo* and *Populus tremula x alba*. A shift from symplastic to apoplastic loading was reported for melon after infection with cucumber mosaic virus (Gil *et al.*, 2011). The tree species *Populus tremula x alba* is associated with the passive phloem-loading mode. Several SUTs have been cloned from this species (Payyavula *et al.*, 2011). PtaSUT4 is localized in the tonoplast of MCs and could facilitate symplastic loading of the minor vein companion cells by modulating cytosolic sucrose concentrations in the nearby mesophyll. *PtaSUT3* is a Group II SUT which is associated with apoplastic transport and transcripts were observed in minor veins of source leaves. By down-regulating the tonoplast PtaSUT4 the level of the Group II PtaSUT3 was increased in mature leaves of poplar (Payyavula *et al.*, 2011). Such morphological and gene expression data in particular point to the heterogeneity in SE-CCCs in minor veins and phloem-loading strategies. These different mechanisms may confer advantages under various environmental conditions.

## Conclusion

Heterologous expression of *FeSUT1* in yeast showed that it codes for a functional SUT. The *K*_m_ value of FeSUT1 was typical for Group II SUTs. Immunolocalization of FeSUT1 revealed that this transporter is expressed in the transport phloem and in the minor veins of *F. excelsior*. In the transport phloem FeSUT1 is involved in the retrieval of sucrose into the large SEs. In minor veins phloem loading of sucrose in *F. excelsior* is at least partly mediated by the activity of FeSUT1 in addition to symplastic phloem loading. Probably during different growing stages or seasons, the proportion of apoplastic and symplastic phloem loading varies depending on the physiological requirements or environmental conditions. This feature could provide flexibility in regard to environmental changes.

## Supplementary data

Supplementary data are available at *JXB* online.

Supplementary Fig. S1. Sequence of the full-length cDNA of *FeSUT1* encoding the Group II SUT FeSUT1 from *F. excelsior*.

Supplementary Data

## Abbreviations:

BSC: bundle sheath cell
CC: companion cell
IC: intermediary cell
FW: fresh weight
MC: mesophyll cell
qPCR: quantitative polymerase chain reaction
RFO: raffinose oligosaccharide
SE: sieve element
SE-CCC: sieve element-companion cell complex
SUT: sucrose transporter.
