# Supplementary material for: Characterization, localization, and seasonal changes of the sucrose transporter FeSUT1 in the phloem of Fraxinus excelsior
Source: J Exp Bot. 2015 May 28;66(15):4807–19. doi: 10.1093/jxb/erv255 (PMC4507781; doi:10.1093/jxb/erv255)
Supplement: Supplementary Data [file supp_66_15_4807__index.html]

Characterization, localization, and seasonal changes of the sucrose transporter FeSUT1 in the phloem of Fraxinus excelsior — Characterization, localization, and seasonal changes of the sucrose transporter FeSUT1 in the phloem of Fraxinus excelsior — Supplementary Data 

# Characterization, localization, and seasonal changes of the sucrose transporter FeSUT1 in the phloem of *Fraxinus excelsior*

## Supplementary Data

Data files

- Supplementary Data - Supplementary Data
