# Supplementary material for: Characterization, localization, and seasonal changes of the sucrose transporter FeSUT1 in the phloem of Fraxinus excelsior
Source: J Exp Bot. 2015 May 28;66(15):4807–19. doi: 10.1093/jxb/erv255 (PMC4507781; doi:10.1093/jxb/erv255)
Supplement: Supplementary Data [file supp_erv255_jexbot146589_file001.pdf]

Journal of Experimental Botany

Characterization and localization of the sucrose transporter FeSUT1 in the phloem of *Fraxinus excelsior*.

Soner Öner-Sieben, Christine Rappl, Ruth Stadler, Norbert Sauer, Gertrud Lohaus

Supplementary data

```
1      CACTCTTTTCATGTAAATTTACTGCATATACATATAGTGAGAGAAATCTATACATACACAATTTCTAAAGTGAGAGAGTG    80

81     AAGAGTTAAATTATATATCCATGGAGGTTGATGGAAGCAAATTGAGTACTATTGTGCCAGAACCTCCACTTACGCCACCG    160
1                          M  E  V  D  G  S  K  L  S  T  I  V  P  E  P  P  L  T  P  P     20
161    CAAGAGCCGCTGAGAAAACTCATTCCGGTGGCGGCCATAGCAGCGGGGGTGCAATTCGGGTGGGCCCTTCAGCTCTCCTT    240
21     Q  E  P  L  R  K  L  I  P  V  A  A  I  A  A  G  V  Q  F  G  W  A  L  Q  L  S  L     47
241    GTTGACTCCGTACGTGCAGCTTCTGGGTATTCCCCATACCTGGGCTGCCTTCATCTGGCTGTGTGGCCAGTCTCCGGCT    320
48     L  T  P  Y  V  Q  L  L  G  I  P  H  T  W  A  A  F  I  W  L  C  G  P  V  S  G  L     74
321    TGTTCGTGCAGCCGATTGTTGGCTACTACAGTGACAATTGCTCCTCCCGCTTTGGGCGGCGCCGCCCCTTTATCGCCGCC    400
75      F  V  Q  P  I  V  G  Y  Y  S  D  N  C  S  S  R  F  G  R  R  R  P  F  I  A  A     100
401    GGTGCCGCACTTGTCGCTGTCGCCGTTTTCCTAATTGGATTTGCCGCGGATATTGGTCATGCCTCCGGGGACCCCCTGGG    480
101    G  A  A  L  V  A  V  A  V  F  L  I  G  F  A  A  D  I  G  H  A  S  G  D  P  L  G     127
481    CAACGACGGCAAGACGGCGAAGCCCCGTGCCATTGCTGTGTTTGTCGTCGGATTTTGGATCCTTGACGTGGCCAACAACA    560
128     N  D  G  K  T  A  K  P  R  A  I  A  V  F  V  V  G  F  W  I  L  D  V  A  N  N  M    154
561    TGTTACAGGGACCTTGCAGAGCCCTGTTGGCTGATCTCTCTGGTGGAAATGCTCAGAAAATGAGGACTGCAAATGCCTTT    640
155     L  Q  G  P  C  R  A  L  L  A  D  L  S  G  G  N  A  Q  K  M  R  T  A  N  A  F     180
641    TTTTCTTTCTTCATGGCGGTGGGGAGCGTGCTCGGTTATGCAGCTGGATCATACACTCACTTGTTCAAAATATTTCCTTT    720
181    F  S  F  F  M  A  V  G  S  V  L  G  Y  A  A  G  S  Y  T  H  L  F  K  I  F  P  F     207
721    TACCAAAACAAAAGCCTGTGATGTGTATTGTGCAAATCTCAAGAGCTGTTTTTTCCTCTCAATATTTCTCTTGTTGACTC    800
208     T  K  T  K  A  C  D  V  Y  C  A  N  L  K  S  C  F  F  L  S  I  F  L  L  L  T  L    234
801    TAACAATATTGGCTTTGACTATTGTGCGTGAAACGGCCTTTGTCCCTTCACAGGCGGAGCCACCAGAAAACATAGGTCAT    880
235     T  I  L  A  L  T  I  V  R  E  T  A  F  V  P  S  Q  A  E  P  P  E  N  I  G  H     260
881    GGGGTGGTTAAGAAGAGGAAAATACCAGTTTTGGGGGAGTTATTTGGTGCATTGAAGGGCTTACCTAGGCCAATGAGGGC    960
261    G  V  V  K  K  R  K  I  P  V  L  G  E  L  F  G  A  L  K  G  L  P  R  P  M  R  A     287
961    TTTGCTCTTGGTGACTTGTTTAAATTGGATTGCTTGGTTCCCATTCTTGTTGTTCGACACTGATTGGATGGGGAAGGAGG    1040
288     L  L  L  V  T  C  L  N  W  I  A  W  F  P  F  L  L  F  D  T  D  W  M  G  K  E  V    314
1041   TGTACGGAGGGAAGGTGGGGGTAGGGAAGTTGTACAACCAAGGGGTGCGTGCAGGGGCACTCGGGCTGATGCTGAGTGCT    1120
315     Y  G  G  K  V  G  V  G  K  L  Y  N  Q  G  V  R  A  G  A  L  G  L  M  L  S  A     340
1121   GTGGTGTTGGGGTGCGCCTCCCTCGCTCTTGAGTTATTGGCGCGTGGACTTGGTGGGGTGAAGCGGCTTTGGGGTGTTGC    1200
341    V  V  L  G  C  A  S  L  A  L  E  L  L  A  R  G  L  G  G  V  K  R  L  W  G  V  A     367
1201   CAACTTCTTGTTAGCCATTTGCTTGGCCATGACCGTCTTGATAACCAAATTGGCGCAGTCTACACGCCGTCATGCCATCC    1280
368     N  F  L  L  A  I  C  L  A  M  T  V  L  I  T  K  L  A  Q  S  T  R  R  H  A  I  H   394
1281   ATAATGGTGATGCTAATCCACCACCGGCCACTGGTGTCAAGGTCGGTGCTTTGGCCCTCTTTGCTATTCTTGGCATTCCT    1360
395     N  G  D  A  N  P  P  P  A  T  G  V  K  V  G  A  L  A  L  F  A  I  L  G  I  P     420
1361   CAAGCGGTGACTTTCAGCATCCCATTTGCTCTAGCGTCTATATTTTCTAGTGATTCTGGGGCAGGACAAGGTCTGTCACT    1440
421    Q  A  V  T  F  S  I  P  F  A  L  A  S  I  F  S  S  D  S  G  A  G  Q  G  L  S  L     447
1441   CGGAGTTCTAAATCTTGCTATAGTTGTACCACAGATGGTGGTATCAGTGCTGAGTGGACCGTGGGATGCCTTGTTTGGAG    1520
448     G  V  L  N  L  A  I  V  V  P  Q  M  V  V  S  V  L  S  G  P  W  D  A  L  F  G  D    474
1521   ACGGTAACTTACCGGCATTCGTTGTGGGCGCCGTGGCAGCTGCGGCTAGCGGGATATTTGCACTCACCCTACTTCCATCT    1600
475     G  N  L  P  A  F  V  V  G  A  V  A  A  A  A  S  G  I  F  A  L  T  L  L  P  S     500
1601   TTACCTTCCGATGTTTCACCAGCCAAGGCCATGAGCGCCGGAGGATTTCATTGAAATATCGATGCATCCTAAGCTAGATA    1680
501    L  P  S  D  V  S  P  A  K  A  M  S  A  G  G  F  H                                  517
1681   GTTTGTTTTCCTTCTCCTACCTTGTTTTTGGGTATTGTGATAGTCCTTTCCTTTTTTTTGCCCTTTCGACCGAAACTTCG    1760

1761   GCATTTTTTAGCCAAGGACATCAACTTATGTATAATATAACTGTGATTTTGTAATGTTTGGACCGTCGAACAGAATTGGG    1840

1841   GAGCAAAAAAAAAAAAAAAAAAAAAAAAA                                                       1869
```

Fig. S1. Sequence of the full-length cDNA clone of *FeSUT1* encoding the Group II SUT FeSUT1 from *F. excelsior*. Transmembrane prediction was performed using the MEMSAT3 & MEMSAT-SVM algorithm of the PSIPREP-Server of the University College London (http://bioinf.cs.ucl.ac.uk/psipred/). Predicted transmembrane domains are highlighted in grey. Start and stop codons are underlined.
